# Knowledge mapping and current trends of Warburg effect in the field of cancer

**DOI:** 10.3389/fonc.2023.1264083

**Published:** 2023-10-26

**Authors:** Quan Zhao, Lina Wang, Zongwei Lv, Xia Wang, Zhenqun Xu, Kefeng Wang

**Affiliations:** ^1^ Department of Urology, Shengjing Hospital of China Medical University, Shenyang, China; ^2^ Department of Anesthesiology, Shengjing Hospital of China Medical University, Shenyang, China

**Keywords:** bibliometrics, Warburg effect, VOSviewer, CiteSpace, cancer

## Abstract

**Background:**

Since abnormal aerobic glycolysis was first identified in cancer cells, many studies have focused on its mechanisms. The purpose of this study was to analyze the global research status of the Warburg effect in cancer using bibliometrics.

**Methods:**

Articles published from 01 January 2013 to 31 December 2022 (n=2,067) were retrieved from the Web of Science core collection database and analyzed using VOSviewer and CiteSpace software.

**Results:**

Over the past decade, there was an overall increase in the number of annual publications. China was the most productive country with 790 articles, while the United States received the most citations, with 25,657 citations in total. Oncotarget was the most productive and most cited journal, with 99 articles and 4,191 citations, respectively. International cooperation was common, with the USA cooperating most with other countries. Lactate metabolism, citrate production, and non-coding RNAs related to the Warburg effect have received increasing attention in cancer research. These areas may become future research trends.

**Conclusion:**

The study findings help summarize the research status and hotspots of the Warburg effect cancer, and will inform subsequent research.

## 1 Introduction

When discussing cancer cell metabolism, it is almost inevitable to mention Otto Heinrich Warburg’s pioneering discovery that cancer cells consume much more glucose than their counterparts in normal tissues. Even when oxygen is plentiful, cancer cells metabolize glucose mainly through glycolysis (1–4). This phenomenon is also known as aerobic glycolysis, and has been widely accepted as a metabolic hallmark of cancer (5–8).

The presence of aerobic glycolysis has been demonstrated in mouse fibroblasts (9, 10), rat thymocytes (11), human lymphocytes (12, 13), and other cell types. These findings indicate that this action is very common among the proliferating tissues of multicellular animals. Further investigation revealed that the main reason is that proliferating cells process glucose in this seemingly “inefficient” manner to generate sufficient glycolytic intermediates required for biomass synthesis (14). In addition, abnormal glycolysis rapidly consumes local glucose and increases lactate levels around cancer tissue, leading to significant changes in the immune microenvironment (15–18). Therefore, the mechanisms and signaling pathways that transform normally differentiated cells to an aerobic glycolysis status are important for the understanding and treatment of cancer.

Bibliometrics originated in the early 20th century. It is a quantitative method to evaluate and study the literature of a certain subject (19, 20). Analyzing the retrieved information, including authors, keywords, journals, institutions, countries and references, can provide an overview of developments and trends in the field of interest. VOSviewer and CiteSpace software allow graphical visualization of the retrieved information (21–23). Further and more comprehensive analyses, such as co-citation, can reveal relationships between two articles cited by one or more articles simultaneously.

In this article, we attempt to summarize the research trends and focus of the Warburg effect in cancer. Our aims are to clarify the knowledge of this research and help inform future studies.

## 2 Materials and methods

### 2.1 Data source and search strategy

**Figure 1:**
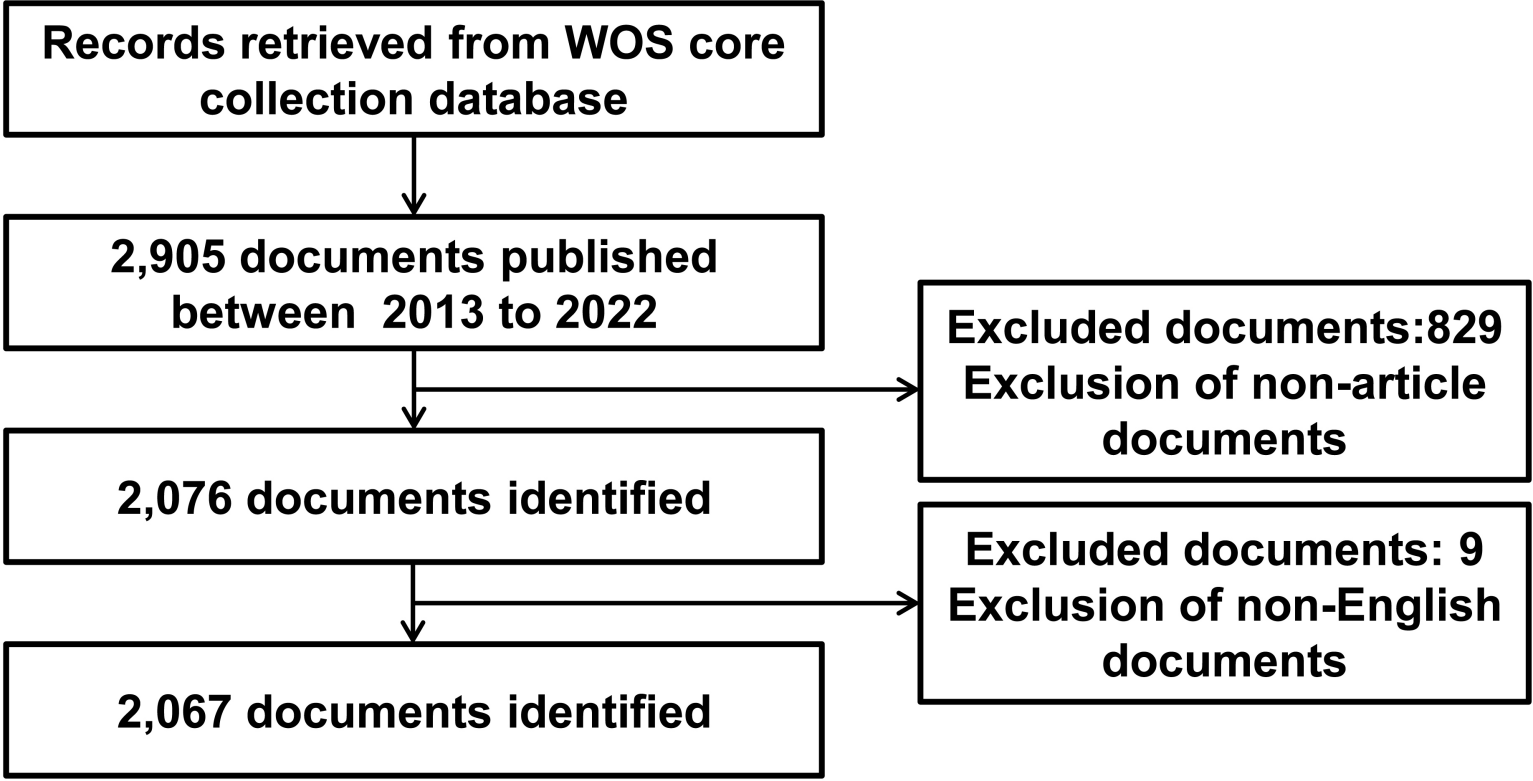
Flow diagram of the literature screening related to Warburg effect in cancer.

We used Web of Science (WOS) core collection database, which is a widely accepted, high-quality database that is recognized as being suitable for bibliometric analysis (24). The search query was shown as follows: TS=((Warburg effect) AND ((carcinoma) OR (cancer) OR (maligan*))). The time span was limited from 2013-01-01 to 2022-12-31. The types of documents were limited to original research articles and the language was limited to English. Eventually, a total of 2,067 articles were selected. The detailed process of data retrieval was shown in **Figure 1**.

### 2.2 Data collection and analysis

The retrieved files were downloaded with full records and cited references in text format. VOSviewer 1.6.18 software was adopted to analyze the basic statistical data, co-citation, and co-occurrence. The results were visualized in network, overlay, and density maps (25–27). In these maps, colors were generated according to certain rules. For the network map, the largest, second, third, and fourth clusters are represented by red, green, blue, and yellow nodes, respectively. As for the overlay map, the color represents the average year of a particular item, ranging from purple to yellow as the average time approached.

CiteSpace v.6.1 was used to analyze burstness and generate a timeline view of keywords (21). In the timeline map, items arranged in the same clusters are shown in each horizontal line. The color of lines connecting the nodes represent the average year of co-occurrence. As time approaches, the color changes from purple to red. The rings around nodes represent the frequency of occurrence in each corresponding year. The ring thickness indicates the frequency of the use of the keyword in that year, with a thicker ring indicating more frequent use.

Microsoft Excel 2010 was used to compile the basic descriptive information and calculate the fitting function of the annual publications.

## 3 Results

### 3.1 Descriptive statistics

**Figure 2:**
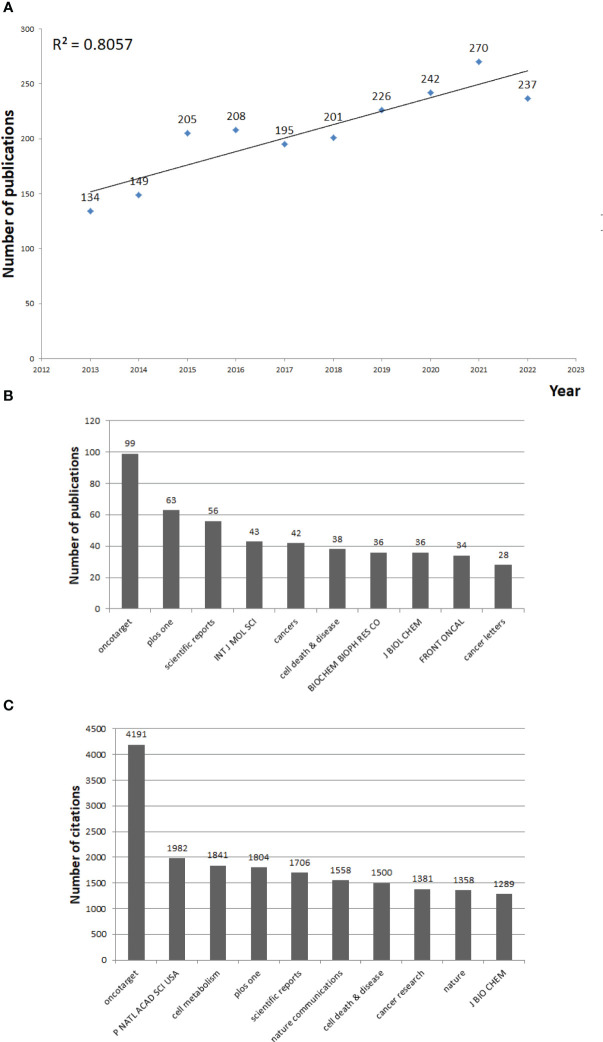
Annual trends of publications and citations on Warburg effect in cancer between 2013 and 2022. **(A)** Number of annual publications between 2013 and 2022. **(B)** Top 10 most productive journals. **(C)** Top 10 most cited journals.

The search performed over the past decade identified 2,067 articles (**Figure 2A**). The highest number of publications was in 2021 and the lowest in 2013. The annual publications displayed a rising trend, indicating that studies on the Warburg effect have received increasing attention globally. A total of 597 journals published articles on the Warburg effect in the past decade; 91 journals published at least five articles. The top 10 journals in terms of output and citations are shown in **Figures 2B, C**. The most productive and cited journals were Oncotarget, while the second most productive and cited journals were PLOS One and Proceedings of The National Academy of Sciences of The United States of America, respectively.

### 3.2 Bibliometric analysis of publications and citations

**Figure 3:**
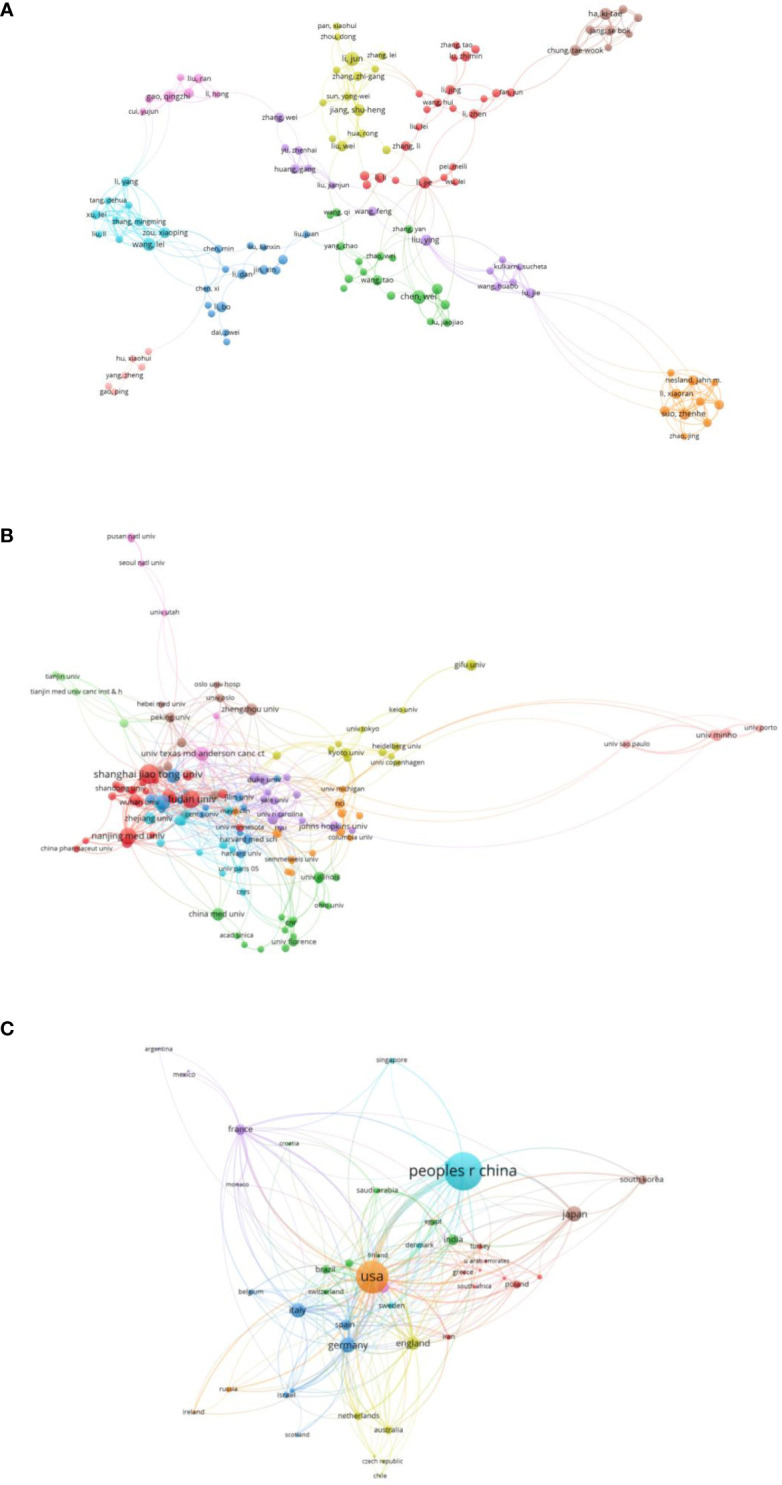
Bibliometric analysis of publications on Warburg effect in cancer. **(A)** Bibliometric analysis of authors on Warburg effect in cancer. **(B)** Bibliometric analysis of institutions on Warburg effect in cancer. **(C)** Bibliometric analysis of countries on Warburg effect in cancer.

Network visualization maps of cited authors, institutions, and countries were generated using VOSviewer, as shown in **Figures 3A–C**. The top 10 most productive authors, institutions, and countries are listed in **Tables 1**–**3**. Shanghai Jiao Tong University was the most productive and most cited institution, with a total of 70 papers that were cited 2,576 times (**Table 2**). Researchers from the United States published 590 articles that were cited 25,657 times, making it the most cited country (**Table 3**).

**Table 1:** Top 10 most active authors related to Warburg effect in cancer.

Rank	Author	Documents	Citations	Average citations
1	Akao,Yukihiro	16	546	34
2	Baltazar,Fatima	16	446	28
3	Taniguchi,Kohei	15	528	35
4	Sugito,Nobuhiko	13	427	33
5	Li,Jun	13	556	43
6	Chen,Wei	12	474	40
7	Shinohara,Haruka	11	401	36
8	Wang,Lei	11	386	35
9	Ha,Ki-tae	10	136	14
10	Granja,Sara	10	245	25

**Table 2:** Top 10 most active institutions related to Warburg effect in cancer.

Rank	Institutions	Documents	Citations	Average citations
1	Shanghai Jiaotong University	70	2,576	37
2	Fudan University	56	1,754	31
3	Nanjing Medical University	51	2,055	40
4	Chinese Academy of Sciences	39	1,964	50
5	University of Texas MD Anderson Cancer Center	34	1,918	56
6	Sun Yat Sen University	33	1,125	34
7	Sichuan University	29	1,302	45
8	China Medical University	28	492	18
9	Zhejiang University	26	749	29
10	Zhengzhou University	24	594	25

**Table 3:** Top 10 most active countries related to Warburg effect in cancer.

Rank	Countries	Documents	Citations	Average citations
1	People’s Republic of China	790	20,837	26
2	The USA	590	25,657	43
3	Japan	131	3,494	27
4	Germany	126	2,647	21
5	Italy	116	3,812	33
6	England	89	2,427	27
7	Canada	76	3,283	43
8	France	66	2,589	40
9	India	62	785	13
10	Spain	60	1,867	31

### 3.3 Bibliometric analysis of co-authorship

#### 3.3.1 Authors

**Figure 4:**
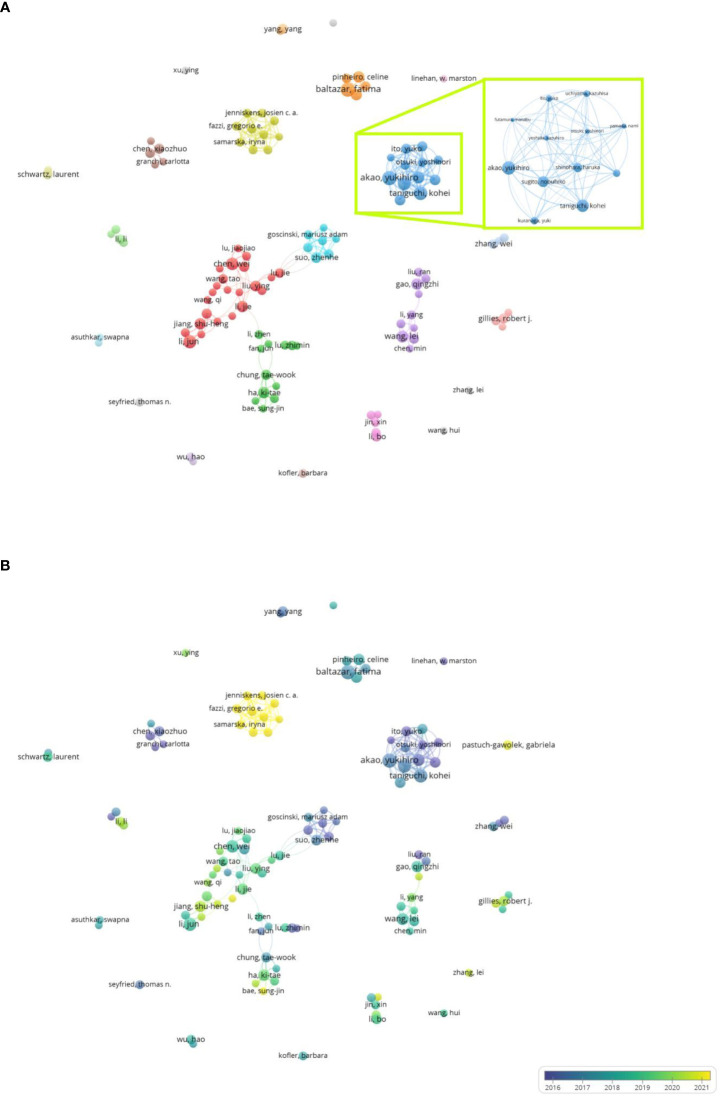
Bibliometric analysis of the co-authorship of authors on Warburg effect in cancer. **(A)** Network visualization map of authors collaboration on Warburg effect in cancer. **(B)** Overlay visualization map of authors collaboration on Warburg effect in cancer.

Out of 13,647 authors, 116 with at least five articles were selected for co-authorship analysis. In the network map of **Figure 4A**, the clusters at the top represent the groups of collaborating authors. The largest cluster mainly included three authors with more than seven articles: Jiang Shuheng and Li Jun from Shanghai Jiaotong University and Chen Wei from Xi’an Jiaotong University. The research group of Shanghai Jiaotong University published the most cited article in July 2017 entitled “Increased Serotonin Signaling Contributes to the Warburg Effect in Pancreatic Tumor Cells Under Metabolic Stress and Promotes Growth of Pancreatic Tumors in Mice”. In this article, the authors demonstrated that *5*-*hydroxytryptamine* (5-HT) increased the expression of hydroxytryptamine receptor 2B in pancreatic ductal adenocarcinoma tissue and cells, thereby facilitating tumor glycolysis and growth (28).

The most productive author in this field was Yukihiro Akao of Gifu University, shown in the blue cluster. His collaborators included Sugito Nobuhiko from Gifu University, and Taniguchi Kohei from Osaka Medical and Pharmaceutical University. These researchers have focused on mechanisms related to microRNAs (miRNAs). In their most cited article, the authors described that miR-124 induced the expression of the pyruvate kinase M (PKM) isoform to catalyze the conversion of PKM2 to PKM1, thereby altering glycolytic metabolism and suppressing human colorectal cancer tumors through a feedback cascade involving polypyrimidine tract-binding protein 1 (PTB1), PKM1, and PKM2 (29).

The overlay map depicted in **Figure 4B** reveals the recent activity of a group of authors, including Gregorio E. Fazzi, Colinda C.J.M. Simons, and Kim M. Smits. Most of their articles on the Warburg effect were published in 2022. The findings suggest that the Warburg effect was related to body mass index in adolescents and colon cancer in men, and to energy restriction during the recession with colon cancer in women (30).

#### 3.3.2 Institutions/countries

Of 2,478 institutions, 87 with more than 10 publications were selected for co-authorship analysis. The top five institutions with the highest link strength were Shanghai Jiaotong University, Chinese Academy of Sciences, Fudan University, Nanjing Medical University, and University of Minho. One of the most influential articles was published in March 2016 by Shanghai Jiaotong University in collaboration with the University of Texas MD Anderson Cancer Center. The article described that the protein kinase phosphoglycerate kinase 1 (PGK1) coordinates the glycolysis and tricarboxylic acid cycles, indicating the important role of PGK1 in tumorigenesis (31).

**Figure 5:**
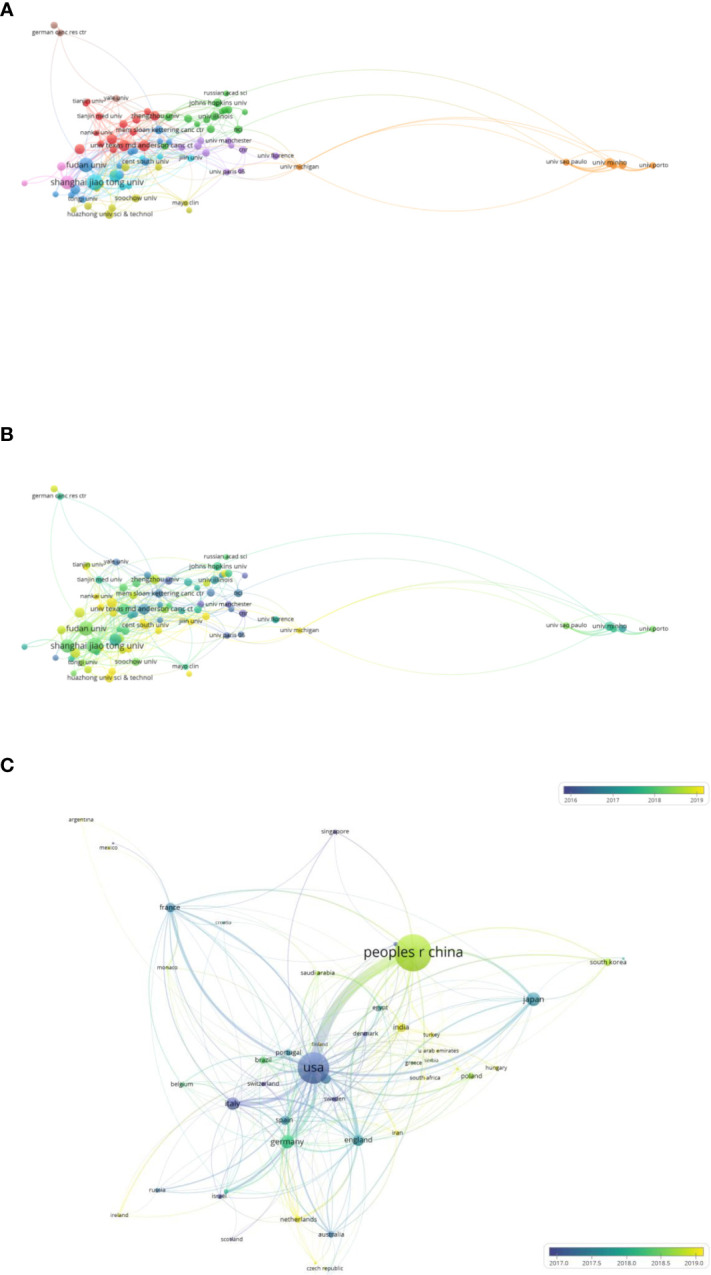
Bibliometric analysis of the co-authorship of institutions/countries on Warburg effect in cancer. **(A)** Network visualization map of institutions collaboration on Warburg effect in cancer. **(B)** Overlay visualization map of institutions collaboration on Warburg effect in cancer. **(C)** Overlay visualization map of countries collaboration on Warburg effect in cancer.

The network map of **Figure 5A** reveals that almost all the clusters comprised institutions from different countries. For instance, the red cluster consisted of University of Texas MD Anderson Cancer Center, Zhengzhou University, Nankai University, and other institutions, indicating that international collaborations in this field were common.

In recent years, several institutions have taken a keen interest in studying the Warburg effect. As evident from the overlay map shown in **Figure 5B**, most were Chinese universities, including Nankai University, Central South University, Jilin University, Huazhong University of Science and Technology, and others.

The overlay map of **Figure 5C**, reveals the strong linkage between the USA and most countries. This indicates that USA was usually the core member of these international collaborations, with the highest link strength of 380. The USA had the thickest connecting line between China, the most productive country, representing the largest scale of global cooperation. The average publication date of western developed countries, such as the USA, France, England and Italy, was approximately 2017. The average publication date of most Asian countries was approximately 2019. These findings indicate that more attention and resources have been devoted to the study of Warburg effect in cancer in Asia. In addition, strong interest in this area was evident in researchers from several European countries, including Ireland, Netherlands, Czech Republic, and others.

### 3.4 Bibliometric analysis of co-citation

**Figure 6:**
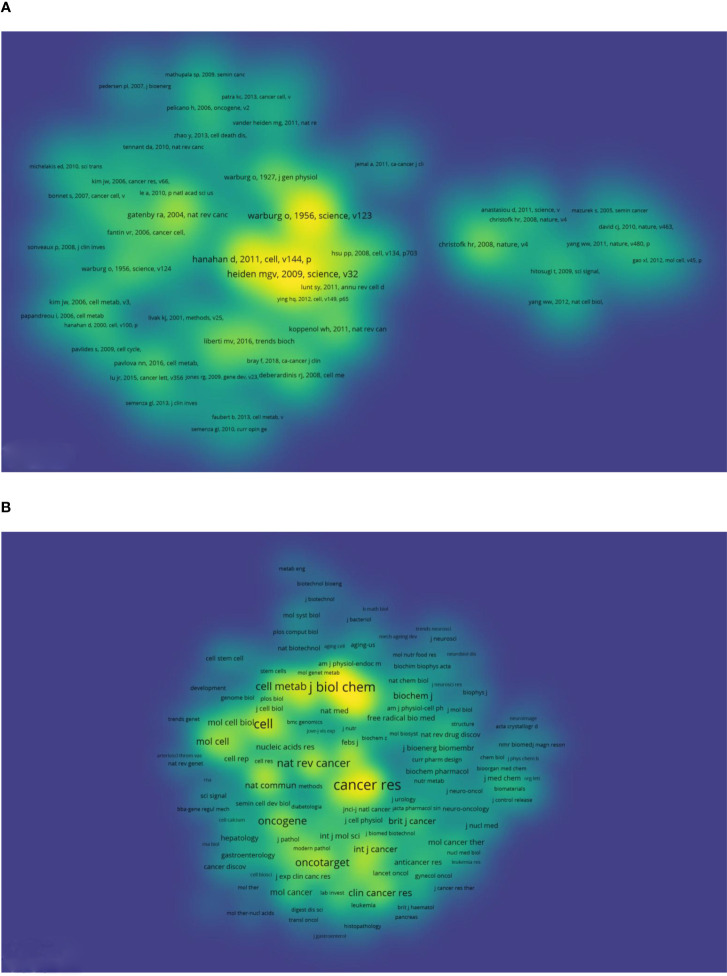
Bibliometric analysis of the co-citation on Warburg effect in cancer. **(A)** Density visualization map of co-cited references on Warburg effect in cancer. **(B)** Density visualization map of co-cited journals on Warburg effect in cancer.

Co-citation analysis reflects the development or changes in a certain area, and can be used to assess the research status and development frontiers. In this study, we set the threshold at 40 and selected 71 articles. The results are depicted as a density map in **Figure 6A**. The top 10 highest co-cited documents are listed in **Table 4**. Most of these highly co-cited documents were reviews. The article with the most co-citations was a review by Matthew G. Vander Heiden MGV in Science in 2009, which was cited 702 times. Six articles were published by American authors, and two by Swiss authors.

**Table 4:** Top 10 highest co-cited documents related to Warburg effect in cancer.

Rank	Article title	Author	Country	Year	Journal	Citations
1	Understanding the Warburg Effect: the metabolic requirements of cell proliferation	Heiden MGV	The USA	2009	Science	702
2	On the origin of cancer cells	Warburg O	The USA	1956	Science	598
3	Hallmarks of cancer: the next generation	Hanahan D	Switzerland	2011	Cell	492
4	Why do cancers have high aerobic glycolysis?	Gatenby RA	The USA	2004	Nature Reviews Cancer	228
5	Regulation of cancer cell metabolism	Cairns RA	Canada	2011	Nature Reviews Cancer	217
6	The M2 splice isoform of pyruvate kinase is important for cancer metabolism and tumour growth	Christofk HR	The USA	2008	Nature	207
7	The Warburg Effect: How Does it Benefit Cancer Cells?	Liberti MV	The USA	2016	Trends in Biochemical Sciences	187
8	Otto Warburg’s contributions to current concepts of cancer metabolism	Koppenol WH	Switzerland	2011	Nature Reviews Cancer	184
9	THE METABOLISM OF TUMORS IN THE BODY	Warburg O	Germany	1927	Journal of General Physiology	182
10	On respiratory impairment in cancer cells	Warburg O	The USA	1956	Science	145

The density map of co-cited references in **Figure 6B** depicts several highlighted journals. There were three journals with more than 2,500 co-citations. These journal were Cancer Research with 3,535 co-citations and an impact factor (IF)/journal citation reports (JCR) partition of 11.2/Q1; Journal of Biological Chemistry with 2,952 co-citations and an IF/JCR partition of 4.8/Q2; and Science with 2,926 co-citations and an IF/JCR partition of 56.9/Q1.

### 3.5 Bibliometric analysis of keywords

Keywords summarize the core of an article. The hotspots and trends in this research field can be revealed by keywords analysis. The findings from bibliometric analysis programs are subsequently discussed in the next section based on three visualized maps.

#### 3.5.1 Co-occurrence

Out of 7,264 keywords retrieved, we used VOSviewer to generate co-occurrence maps, of which 115 keywords appeared at least 30 times. In addition to the main topic (the Warburg effect), keywords that appeared more than 200 times were metabolism, expression, cancer, glycolysis, growth, apoptosis, cells, hypoxia, aerobic glycolysis, proliferation, and inhibition.

**Figure 7:**
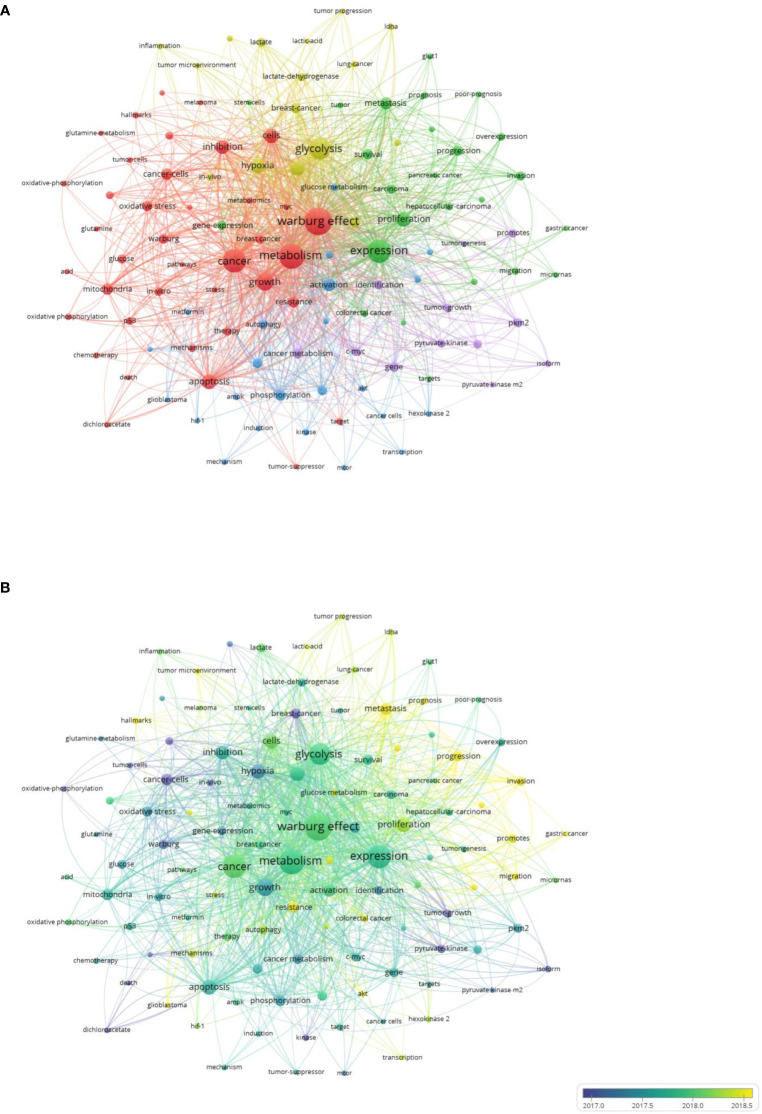
Bibliometric analysis of the co-occurrence of keywords on Warburg effect in cancer. **(A)** Density visualization map of high frequency keywords on Warburg effect in cancer. **(B)** Oerlay visualization map of high frequency keywords on Warburg effect in cancer.

The network map of **Figure 7A** reveals that 115 keywords were arranged into five clusters by the algorithm. The red cluster was mainly based on basic research and included 39 items, such as metabolism, cancer, growth, apoptosis, and inhibition, and others. The green cluster was generally clinical and contained 25 items, including expression, progression, proliferation, metastasis, and others. The blue cluster involved metabolism. The 23 cluster items included activation, phosphorylation, glucose metabolism, autophagy, and others. The yellow cluster involved the tumor microenvironment. It included 16 items, such as glycolysis, hypoxia, lactate, and others. Finally, the purple cluster focused on specific genes and enzymes. This cluster contained 12 items, such as c-myc, pkm2, pyruvate kinase, and others.

In the overlay map of **Figure 7B**, several keywords were nearly pure yellow, indicating that they have gained more attention since 2018. These words included tumor microenvironment, metastasis, progression, prognosis, invasion, migration, resistance, akt, and some specific cancers. The findings indicate two aspects. First, current research on the Warburg effect of cancer is mainly focused on the metastatic stage of cancer. Second, basic studies in this field are being combined with clinical medicine.

#### 3.5.2 Timeline map

We used CiteSpace software to generate a timeline map. All the keywords were distributed into 12 clusters, represented by each horizontal line. The same cluster was set to share labels of the same color. We chose nodes of the tree ring type; the color bands around each node represented the number of occurrences of this keyword in each corresponding year. For instance, the node “oral squamous cell carcinoma” was mainly composed of green bands, indicating that this keyword appeared frequently between 2018 and 2020. The color of connecting lines between nodes was set to reflect the average year of co-occurrence.

**Figure 8:**
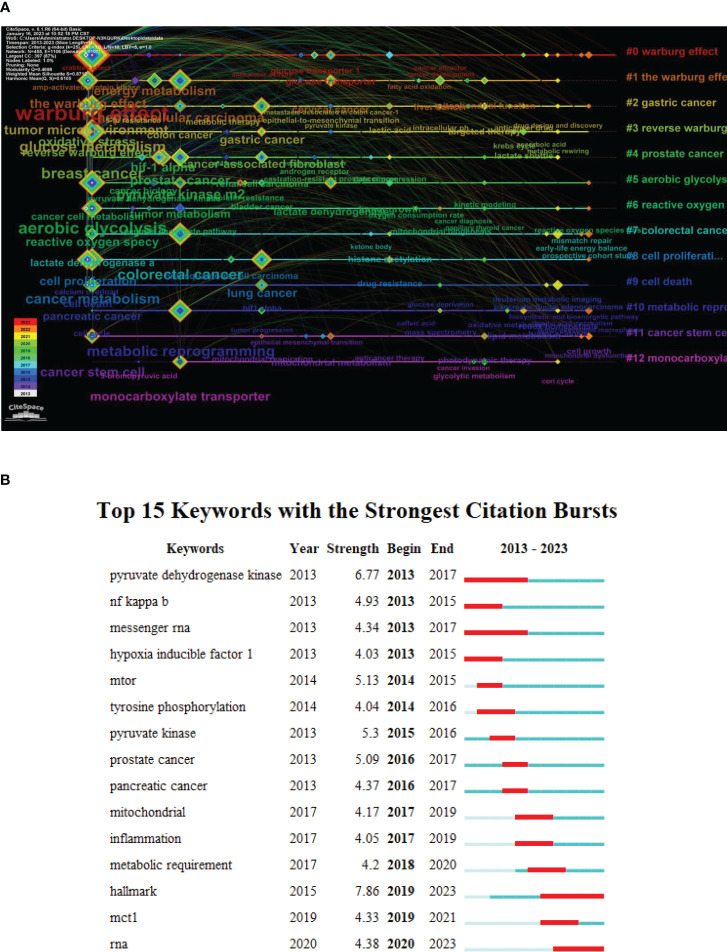
Bibliometric analysis of the timeline map and burstness of keywords on Warburg effect in cancer. **(A)** Bibliometric analysis of the timeline map of keywords on Warburg effect in cancer. **(B)** Bibliometric analysis of the burstness of keywords on Warburg effect in cancer.

From **Figure 8A**, we can infer the trends of the research. Some nodes had almost no orange or red bands, such as hypoxia inducible factor-1α, cancer associated fibroblasts, lactate dehydrogenase A, and epithelial mesenchymal transition, indicating that these aspects have become less studied in recent years. The nodes of glucose transporter, reactive oxygen species, mismatch repair, mitochondrial metabolism, and mitochondrial dysfunction were composed of thick orange or red bands, implying that these nodes may be the research hotspots in this field.

#### 3.5.3 Analysis of burstness

The CiteSpace algorithm detects bursts of research topics. We found 15 keywords with strong citation bursts (**Figure 8B**). The occurrence of burst words was evenly distributed over the past decade, with the strongest burst word being “hallmark”. Three burst words lasted 4 years: “pyruvate dehydrogenase kinase”, “messenger RNA”, and “hallmark”. The continuation of “RNA” from 2020 to the present may indicate that cancer cell metabolism related to RNA is receiving more attention.

## 4 Frontiers in researches

Based on the above bibliometric analysis, we summarized several research frontiers and hotspots of Warburg effect in the field of cancer. These are described below.

### 4.1 Lactate metabolism

Lactate was previously thought to be a byproduct of glycolysis in tumor cells. However, in recent years lactate has been further recognized as an important fuel and signaling agent (32). In addition, large amounts of lactate also contribute to acidification of the tumor microenvironment, which eventually facilitates tumor metastasis, angiogenesis, and immunosuppression (33). Due to the activation of the Warburg effect, cancer cells will produce a large amount of lactate, resulting in cytoplasmic acidification and slow metabolism. In this case, cancer cells need a way to eliminate excessive lactate (34, 35).

Lactate is a weak acid that is hydrophilic and cannot pass directly through the cell membrane. Excess lactate is excreted through monocarboxylate transporters (MCTs). Many cancers share the common feature of overexpressing lactate transporters, making MCTs a possible target for cancer therapy (36–39). In 2018, articles described two relevant compounds. One was AR-C155858, a MCT inhibitor that was considered an immune modulator. The other novel compound was BAY-8002 (40). BAY-8002 is effective in inhibiting hematopoietic tumor cells, particularly diffuse large B-cell lymphoma cells, as well as some subgroups of solid tumors (41).

### 4.2 Citrate production

In normally differentiated cells, pyruvate produced by glycolysis is consumed by mitochondria. In proliferating cells, due to the Warburg effect, pyruvate is distributed to mitochondrial oxidative phosphorylation, resulting in a decrease of citrate from the tricarboxylic acid cycle. There is evidence that citrate concentration is reduced in many different cancer cells. Since citrate is a donor of acetyl-coA, lowering citrate maintains glycolysis by reducing cytoplasmic acidity and facilitates the deacetylation of proteins, thus helping cells resist apoptosis and epigenetic changes (42–44).

Intraperitoneal injection of citrate reported significantly inhibited the growth of gastric cancer in mice, suggesting that citrate may be a suitable treatment for abdominal tumor (42, 45). Citrate also enhances the *in vitro* effects of platinum-based drugs (46). Drugs designed to increase intracellular citrate concentration have also been developed. For instance, the ATP citrate lyase inhibitor hydroxycitrate has been observed to reduce tumor growth in mice (47).

### 4.3 Non-coding RNAs

Because it is widely accepted that genomic instability and consequent genetic diversity are key to many features of cancer (48), many researchers have focused on the molecular mechanisms upstream of enzymes and substrates. In recent years, many non-coding RNAs (ncRNA) have been identified and recognized as major mechanisms of epigenetic control, many of which have been proved to be involved in the occurrence and metastasis of many different cancers (49).

MicroRNAs (miRNAs) can affect energy metabolism through a variety of enzymes and transcription factors, and thus play an important role in the Warburg effect. In a highly cited article published in 2018, Li et al. (50) reported that transcription factor sine oculis homeobox 1 (SIX1) promoted glycolysis through histone acetyltransferases and was overexpressed in many cancers. SIX1 was directly repressed by miR-548a-3p, suggesting a possible therapeutic target on miR-548a-3p/SIX1 axis.

## 5 Limitations

There are some limitations in this study. First, to ensure the quality and integrity of the retrieved data, only the literature in the SCI-expanded index of WOS core collection database was searched. This inevitably led to ignoring of the documents in other databases, such as Scoupus. Second, high-quality articles published recently may have not received enough attention so far, resulting in fewer citations and omissions. Finally, when analyzing and interpreting the retrieved data, a comprehensive understanding of the field is required, and subjectivity is unavoidable.

## 6 Conclusion

The purpose of this study is to help researchers understand the current status and trends of the Warburg effect in cancer. Bibliometrics and visualized maps were used to comprehensively analyze the literature published globally in the past decade. According to our statistics, the number of articles published each year showed an upward trend globally. China and the USA accounted for more than half of the publications. Oncotarget was the most productive and most cited journal. International collaboration was common in this field, and the USA collaborated with most countries. Lactate metabolism, intracellular effects of altered citrate concentration, and molecular mechanisms of ncRNA have received increasing attention in recent years. These studies may inform future research trends.

## Data availability statement

The original contributions presented in the study are included in the article/supplementary material. Further inquiries can be directed to the corresponding authors.

## Author contributions

QZ: Data curation, Methodology, Writing – original draft. LW: Resources, Software, Writing – review and editing. ZL: Investigation, Methodology, Software, Writing – review and editing. XW: Resources, Supervision, Writing – review and editing. ZX: Supervision, Writing – review and editing. KW: Conceptualization, Funding acquisition, Visualization, Writing – review and editing.
